# Identification of activating hotspot mutations in acrochordons through next-generation sequencing

**DOI:** 10.1016/j.jdin.2024.08.011

**Published:** 2024-09-16

**Authors:** Justin L. Jia, Jennifer Y. Wang, Warren H. Chan, Julien Lanoue, Hanh Do, Kerri E. Rieger, S. Tyler Hollmig, Kavita Y. Sarin

**Affiliations:** a Department of Dermatology, Stanford University School of Medicine, Stanford, California; b Department of Medicine, Baylor College of Medicine, Houston, Texas; c Department of Dermatology, University of Vermont Medical Center, Burlington, Vermont; d Department of Pathology, Stanford University School of Medicine, Stanford, California; e Division of Dermatology, Dell Medical School, University of Texas, Austin, Texas

**Keywords:** acrochordon, bioinformatics, dermatology, genetics, hotspot mutations, next-generation sequencing, personalized medicine, skin, skin tag, whole exome sequencing

*To the Editor:* Acrochordons or skin tags have been associated with insulin resistance, hormonal dysregulation, and genodermatoses.^1^ Their increased presentation in certain genodermatoses, such as Birt-Hogg-Dubet syndrome and PTEN hamartoma syndrome, suggest that genetic factors may be implicated in pathogenesis, though the exact etiology remains unclear. We thus sought to identify somatic mutations associated with acrochordons through both wide-exome sequencing (*n* = 5) and targeted next-generation sequencing (*n* = 21). Methods are detailed in Supplemental Material, available via Mendeley at https://data.mendeley.com/datasets/gjffv4c3zw/1.

Whole-exome sequencing was successfully completed on 5 acrochordon samples alongside 4 matched saliva controls (1 was unavailable). After excluding potential germ line variants, mutations in *FGFR3* were identified in 3 of 5 acrochordon samples (Table I). Two demonstrated the hotspot mutation R248C with variant allele frequencies (VAF) of 35% and 22.7%, and another demonstrated a S371C activating mutation (VAF 8.2%). Activating mutations in PIK3CA, HRAS, KRAS, and BRAF were also noted in specimens.

**Table I:** Mutations identified by whole exome sequencing of acrochordons

Sample (sex/age)	Location	FGFR3	PIK3CA	KRAS	HRAS	BRAF
1 (M/72)	Left upper chest	***R248C*** (***22***.***7%***)				
2 (F/66)	Neck	***R248C*** (***35%***)	***E545K*** (***54***.***2%***)			
3 (F/63)	Bilateral eyelids	*S371C* (*8*.*2%*)		***G12D*** (***6***.***2%***)	*K117N* (*4*.*2%*)	
4 (M/51)	Back					***V600E*** (***13***.***6%***)
5 (F/48)	(not available)					

Fresh tissue samples were submitted for whole exome sequencing alongside matched saliva controls. Specific gene mutations are listed with their variant allele frequencies (%). Known activating mutations are italicized, and hotspot mutations are bolded.

We sought to validate these findings by performing targeted next-generation sequencing on an additional 21 acrochordon samples. Twelve of 21 specimens demonstrated at least one mutation in *FGFR3*, *PIK3CA*, *HRAS*, *KRAS*, or *BRAF* (Table II), with 10 specimens demonstrating known activating mutations. Four samples demonstrated mutations in *FGFR3*, including 3 at R248 (R248C or R248H), also seen in whole-exome sequencing samples. Ten of 21 contained at least 1 *PIK3CA* mutation, including hotspot mutations E542K and H1047R in 3 samples. One specimen demonstrated only the *HRAS* hotspot mutation Q61L (VAF 33.7%). Additional activating mutations in KRAS, HRAS, and BRAF were found in a handful of specimens with low VAF.

**Table II:** Mutations identified by targeted next-generation sequencing of acrochordons

Sample	FGFR3	PIK3CA	KRAS	HRAS	BRAF
T	***R248C*** (***45%***)	***E542K*** (***28%***)			
F	***R248C*** (***43***.***9%***)	*M1043I* (*1*.*4%*)	A146E (4.5%)∗		
	S371N (1.5%)∗				
J	***R248H*** (***1***.***5%***)	*R108H* (*1*.*2%*)			
C		***H1047R*** (***1***.***1%***)			
S		***H1047R*** (***1***.***6%***)			
G		*R108H* (*5*.*6%*)	***Q61K*** (***1***.***9%***)		
		R375L (3.4%)∗			
H		C378Y (1.2%)∗		***G13S*** (***1***.***3%***)	
R				***Q61L*** (***33***.***7%***)	
E		R357Q (1.6%)∗		***G12S*** (***1***.***3%***)	
B		P539L (1.4%), R93Q (1.1%)∗			*G466E* (*1*.*2%*)
I	G370D (1.1%)∗				
A		R115Q (2.1%)∗			

Formalin-fixed paraffin-embedded (FFPE) tissue blocks of acrochordon specimens were analyzed by next-generation sequencing with the Stanford Solid Tumor Actionable Mutation Panel to identify mutations in common oncogenes. Specific gene mutations are listed with their variant allele frequencies (%). Known activating mutations are italicized, and hotspot mutations are bolded. Nine additional samples did not harbor mutations in these genes and are not listed.

∗ Mutations of unknown functional impact.

We identified activating mutations in *PIK3CA* and genes involved in MAPK signaling, including *FGFR3*, *KRAS*, *HRAS*, and *BRAF*. Common hotspot mutations, such as *FGFR3* R248C and *PIK3CA* E342K and E345K, were found at high allele fractions in several specimens and have been described in other benign cutaneous neoplasms. For example, seborrheic keratoses often harbor activating mutations in *FGFR3* and *PIK3CA*.^2^^,^^3^ Somatic *HRAS* and *KRAS* mutations have been implicated in the development of nevus sebaceous and epidermal nevi.^4^ Although *BRAF* mutations are not typically identified in keratinocytic neoplasms, they are common among benign and malignant melanocytic lesions.

It is unclear whether activating mutations underlie the primary pathogenesis of acrochordons or whether passenger mutations develop secondarily because of increased cell proliferation. In seborrheic keratoses, one study suggested that *FGFR3* mutations promote mild epidermal hyperplasia but are insufficient to drive lesion development, which may require additional genetic, hormonal, or microenvironmental factors.^3^ This explanation may be applicable to acrochordons as well, given the purported role of IGF-1 signaling in their pathogenesis,^5^ and that only some of our samples demonstrated activating mutations in common cutaneous oncogenes. Limitations of our study include limited sample size from a non-genodermatoses patient population, that sequencing of formalin-fixed paraffin-embedded (FFPE) blocks may introduce degradation artifact, and that some mutations with low-VAF may represent passenger mutations.

In summary, acrochordons may also be associated with activating mutations in the MAPK and PI3K-AKT pathways which exist in similar benign cutaneous neoplasms, suggesting a potential interplay between genetic and environmental factors in their pathogenesis. Understanding the genetic basis of acrochordon development may provide insight into disease mechanisms and potential therapeutic targets.

## Conflicts of interest

None disclosed.
